# Three- and Nine-Month Follow-Up of Patients with COVID-19: Clinical, Functional, and Radiological Outcomes

**DOI:** 10.3390/jcm15135202

**Published:** 2026-07-03

**Authors:** Muhammed Değer, Talat Kılıç, Zeynep Ulutaş, Muhammed Said Tan, Hatice Ödümlü, Ayşenur Atila, Hilal Büşra Demir, Büşra Soysaldı, Miraç Karaağaç, Yunus Emre Er, Ozan Akdağ

**Affiliations:** 1 Department of Pulmonology, Faculty of Medicine, Inonu University Turgut Ozal Medical Center, 44280 Malatya, Türkiye; talatkilic2013@gmail.com (T.K.); mstnusmar47@hotmail.com (M.S.T.); hatice.odumlu@inonu.edu.tr (H.Ö.); aysenur.atila@inonu.edu.tr (A.A.); demirhilalbusra@gmail.com (H.B.D.); yunus-emre.er@inonu.edu.tr (Y.E.E.);; 2 Department of Cardiology, Faculty of Medicine, Inonu University Turgut Ozal Medical Center, 44280 Malatya, Türkiye; zeynep.ulutas@inonu.edu.tr (Z.U.); mirac.karaagac@inonu.edu.tr (M.K.)

**Keywords:** COVID-19, long COVID, post-COVID-19, pandemic, pulmonary sequelae

## Abstract

**Background/Objectives:** The acute complications of COVID-19 have been well characterized and are frequently associated with increased mortality. Although substantial knowledge regarding long COVID has accumulated since the beginning of the pandemic, important uncertainties remain regarding the long-term clinical, functional, radiological, and metabolic consequences of SARS-CoV-2 infection. Identification of post-COVID-19 complications is therefore essential for appropriate recognition and management. This study aimed to evaluate the long-term complications of COVID-19 at 3 and 9 months after infection. **Methods:** This prospective study was conducted at Inonu University Turgut Ozal Medical Center. Patients who presented with active post-COVID-19 complaints or for routine follow-up were enrolled. Participants were evaluated at the pulmonology outpatient clinic at 3 and 9 months. At each visit, persistent or new-onset symptoms were assessed, and pulmonary function tests (PFT), the six-minute walk test (6MWT), echocardiography (ECHO), and thoracic computed tomography (CT) were performed as clinically indicated. Patients were stratified into three groups according to the severity of acute illness: outpatient, ward-hospitalized, and ICU-hospitalized. **Results:** A total of 205 patients (120 male, 85 female) were included. Male patients had significantly higher rates of ward and ICU hospitalization than female patients (*p* = 0.006). At 9 months, 85.3% of patients had at least one persistent symptom; dyspnea (69.6%), cough (35.6%), and chest pain (32.5%) were the most common. FVC showed a statistically significant increase between months 3 and 9 (*p* = 0.014), and the 6MWT distance improved significantly (423.56 m vs. 464.10 m; *p* = 0.008). Ground-glass opacity, present in 90.2% of patients at admission, persisted in 44.3% at 9 months (*p* < 0.001). Reticular opacities, pleuroparenchymal bands, and mosaic perfusion patterns increased over time. ICU patients had significantly lower ejection fraction values compared with ward and outpatient groups at 9 months (*p* = 0.046). During follow-up, 13 patients developed pulmonary embolism and 7 developed new-onset diabetes mellitus. **Conclusions:** Despite the well-characterized acute phase, the long-term sequelae of COVID-19 remain a significant clinical challenge. Identification of late complications is critical for reducing morbidity and understanding the long-term societal and healthcare burden of the pandemic. Multidisciplinary long-term follow-up is warranted, particularly for patients who experienced severe acute illness.

## 1. Introduction

COVID-19 has infected more than 700 million people and caused over 7 million deaths worldwide [1]. In parallel, more than 13 billion vaccine doses have been administered globally [2]. The widespread implementation of vaccination programs, together with successive waves of SARS-CoV-2 variants, including the global predominance of the Omicron lineage and its descendants, contributed to increasing population-level immunity through both vaccination and natural infection [3]. As a result, pandemic-related fears have begun to diminish; however, COVID-19 may not be fully eradicated and could eventually become endemic. The severity of disease and case-fatality rate have declined [4], yet as the number of infected survivors continues to grow, prolonged medical and psychological sequelae have become increasingly recognized [5,6].

COVID-19 is primarily transmitted through respiratory particles, including droplets and aerosols generated during breathing, speaking, coughing, and sneezing [7]. Older age and underlying comorbidities—including hypertension, diabetes mellitus, and cardiovascular disease—are associated with more severe disease [8]. Chen et al. demonstrated that elderly male patients progressed more rapidly to acute respiratory distress syndrome (ARDS), representing a life-threatening condition [9]. A meta-analysis of comorbidities in hospitalized patients reported hypertension in 17%, diabetes in 8%, cardiovascular disease in 5%, and pulmonary disease in 2% of cases [10].

This study was designed to investigate the long-term outcomes of patients with PCR-confirmed COVID-19. We hypothesized that COVID-19 may lead to persistent clinical, functional, and radiological sequelae and that these sequelae would differ according to the severity of the acute illness and hospitalization status.

## 2. Methods

### 2.1. Study Design and Ethics

This was a prospective observational study approved by the Clinical Research Ethics Committee of Inonu University Faculty of Medicine (approval date: 3 March 2021; decision number: 31). Written informed consent was obtained from all participants prior to enrollment. Patients with PCR-confirmed COVID-19 were invited to attend follow-up evaluations between 2021 and 2022. Three- and nine-month follow-up assessments were completed by mid-2022.

### 2.2. Patient Population

Patients who presented to Inonu University Turgut Ozal Medical Center for post-COVID-19 complaints or routine follow-up were screened for eligibility. Inclusion criteria were: age ≥ 18 years, PCR-confirmed SARS-CoV-2 infection, and CT findings during the acute phase. Patients were contacted by telephone and invited for follow-up visits at 3 and 9 months after confirmed infection. Patients were stratified into three groups based on acute illness severity: outpatient, ward-hospitalized, and ICU-hospitalized.

At each follow-up visit, presenting symptoms, smoking history, in-hospital treatment details (including corticosteroid and LTOT use), duration of hospitalization, and radiological findings from the acute phase were recorded.

Because this study was conducted in a tertiary referral center, the study population may not be fully representative of the broader population of COVID-19 survivors. Therefore, the findings should be interpreted with consideration of potential referral bias.

### 2.3. Pulmonary Function Tests (PFT)

PFTs were performed according to American Thoracic Society (ATS) criteria [11]. Flow-volume curves were used to derive FEV1, FVC, and FEV1/FVC values. Restrictive pattern was defined as FEV1/FVC > 70% with FVC < 80% predicted. Obstructive pattern was defined as FEV1/FVC < 75% with a compatible flow-volume curve.

### 2.4. Six-Minute Walk Test (6MWT)

The 6MWT was performed in the bronchoscopy unit of the Department of Pulmonology along a 50 m corridor. Patients were instructed to walk at their maximum comfortable pace.

### 2.5. Computed Tomography (CT)

Thoracic CT examinations were performed and reported by radiologists at the Department of Radiology, Inonu University. CT was requested at the discretion of the attending physician based on symptom burden, using non-contrast, contrast-enhanced, or pulmonary embolism protocol as appropriate. The following radiological patterns were evaluated: ground-glass opacity (GGO), interlobular septal thickening, reticular opacity, cystic lesion, consolidation, honeycombing, pleuroparenchymal band formation (PPBF), mosaic perfusion, pulmonary embolism, cavitary lesion, nodular lesion, and pleural effusion. Any single finding, regardless of extent, was considered significant.

### 2.6. Echocardiography (ECHO)

Patients were referred to the Department of Cardiology for cardiac evaluation. All measurements were performed by an experienced cardiologist according to the American Society of Echocardiography guidelines [12]. Pulmonary artery pressure (PAP) and ejection fraction (EF) were recorded.

### 2.7. Statistical Analysis

Statistical analyses were performed using IBM SPSS Statistics for Windows, version 25.0 (IBM Corp., Armonk, NY, USA). Normality of the data was assessed using the Shapiro–Wilk test, histogram distribution, and skewness–kurtosis parameters. The Shapiro–Wilk test was used to determine whether continuous variables followed a normal distribution and to guide the selection of appropriate parametric or non-parametric statistical tests.

Variables with normal distribution are presented as mean ± standard deviation (SD), whereas non-normally distributed variables are presented as median (minimum–maximum). Categorical variables are expressed as frequencies and percentages.

Chi-square and Fisher’s exact tests were used to compare categorical variables between groups. Fisher’s exact test was preferred when expected cell counts were small. Continuous variables with normal distribution were compared using analysis of variance (ANOVA) to assess differences among hospitalization groups, whereas non-normally distributed variables were analyzed using the Kruskal–Wallis test. When overall group differences were significant, post hoc analyses were performed using Tukey’s test following ANOVA and Bonferroni-corrected Mann–Whitney U tests following Kruskal–Wallis analysis to identify the groups responsible for the observed differences.

Repeated-measures general linear model analysis was used to evaluate within-subject changes between the 3- and 9-month follow-up assessments and to determine longitudinal changes in clinical and functional parameters over time.

The selection of statistical tests was based on the distribution characteristics of the data, the type of variables analyzed, and the longitudinal design of the study. Similar statistical approaches have been used in recent post-COVID follow-up studies evaluating long-term clinical, functional, and radiological outcomes [13].

A two-sided *p*-value < 0.05 was considered statistically significant.

## 3. Results

### 3.1. Demographic and Clinical Characteristics

A total of 205 patients (120 male, 85 female) were included. The mean age was 59.0 years (range: 22–88). During the acute phase, 30.7% were managed as outpatients, 40.5% were hospitalized in a general ward, and 28.8% required ICU admission. The mean ward hospitalization duration was 12.79 ± 10.0 days and mean ICU stay was 17.49 ± 20.5 days. Prednisolone was administered to 122 patients (59.5%), and 53 patients (25.9%) received long-term oxygen therapy (LTOT) after discharge. Demographic characteristics and comorbidities are summarized in Table 1.

Male patients had significantly higher rates of ward and ICU hospitalization compared with females (*p* = 0.006). Prednisolone use was significantly associated with hospitalization status (*p* < 0.001), with 91.5% of ICU patients, 65.1% of ward patients, and 22.2% of outpatients receiving prednisolone. LTOT was prescribed to 57.6% of ICU patients and 22.9% of ward patients (*p* < 0.001).

### 3.2. Symptoms

At admission, 97.6% of patients had at least one symptom. At the 3-month visit, 137 of 148 patients (92.6%) reported at least one symptom. At 9 months, 163 of 191 patients (85.3%) still had at least one symptom. Dyspnea, cough, and chest pain were the most common persistent or newly reported symptoms. Detailed symptom data across time points are presented in Table 2.

### 3.3. Radiological Findings

CT involvement was detected in 96.6% of patients at admission, 92.1% at 3 months, and 79.5% at 9 months. Ground-glass opacity, present in 90.2% at admission, persisted in 44.3% at 9 months (*p* < 0.001). Interlobular thickening and consolidation decreased over time; however, reticular opacities, pleuroparenchymal bands, mosaic perfusion patterns, and honeycombing showed an increase compared with baseline. CT findings across time points are presented in Table 3. 

### 3.4. Pulmonary Function and Exercise Capacity

Among patients with paired PFT data (*n* = 61), both FEV1% and FVC% showed statistically significant improvements between months 3 and 9 (*p* = 0.026 and *p* = 0.009, respectively). The FEV1/FVC ratio did not change significantly (*p* = 0.120). The 6MWT distance increased from 423.56 ± 122.7 m at month 3 to 464.10 ± 103.8 m at month 9 (*p* = 0.008). At the 9-month visit, ICU patients had significantly higher FEV1/FVC values compared with other groups (*p* = 0.047), likely reflecting a predominantly restrictive pattern. Functional and cardiac parameters are summarized in Table 4.

### 3.5. Echocardiographic Findings

At 9 months, ICU patients had significantly lower mean EF values (55.88 ± 8.72%) compared with ward (58.49 ± 4.80%) and outpatient groups (57.72 ± 4.92%) (*p* = 0.046). No significant differences were observed in PAP or RV dilation across groups at 9 months. Between-visit comparisons revealed a trend toward EF increase and PAP decrease, but neither reached statistical significance.

### 3.6. CT Findings by Hospitalization Group

At 9 months, CT involvement was significantly more prevalent in ICU (88.5%) and ward patients (85.5%) compared with outpatients (63.6%) (*p* = 0.003). Interlobular thickening was present in 65.4% of ICU patients, 47.8% of ward patients, and 41.8% of outpatients (*p* = 0.041). Reticular opacity, mosaic perfusion, and pleuroparenchymal bands were more frequent in ICU-hospitalized patients (Table 5).

### 3.7. New-Onset Conditions During Follow-Up

Several new conditions were diagnosed during the 9-month follow-up period (Table 6). Pulmonary embolism was the most frequent new diagnosis (*n* = 13; prevalence 6.34%). New-onset diabetes mellitus was confirmed in 7 patients (prevalence 3.4%), with 6 of 7 patients having received prolonged corticosteroid therapy. Lung cancer was detected in 5 patients, fungal infections in 4, and interstitial lung disease in 3. A total of 14 patients died during follow-up; mortality did not differ significantly by hospitalization group (*p* = 0.884).

Pulmonary embolism diagnoses were established in symptomatic patients evaluated because of clinical suspicion. Of the 13 cases, 6 were identified at the initial evaluation, 6 during the 3-month follow-up, and 1 during the 9-month follow-up assessment.

## 4. Discussion

This study prospectively evaluated the clinical, radiological, and functional characteristics of COVID-19 survivors over 9 months, comparing findings with published literature. Key findings include the following: male sex was associated with higher hospitalization and ICU admission rates; greater acute disease severity was associated with more frequent long-term symptoms, functional impairment, and radiological abnormalities; pulmonary function and exercise capacity improved progressively over time; and certain comorbidities—particularly diabetes—were associated with delayed radiological recovery.

Long COVID is defined as a multisystem condition characterized by symptoms persisting or recurring for at least 3 months following SARS-CoV-2 infection [5]. Although vaccine development has reduced mortality and severe disease, the mutation capacity and transmissibility of the virus ensure that long-term effects remain clinically important [3].

In this cohort, male patients had significantly higher ward and ICU admission rates than female patients (*p* = 0.006), consistent with prior studies from the United States [14], Italy [15], and a large U.S. multicenter study demonstrating higher intubation rates, longer hospitalization, and greater in-hospital mortality in male patients [16]. The increased susceptibility of males to severe COVID-19 is thought to be related to hormonal differences, sex-specific immune responses, and comorbidity burden [17].

Persistent symptoms were observed in 85.3% of patients at 9 months, with dyspnea, cough, and chest pain being most prevalent. This is consistent with published studies in which fatigue and dyspnea dominated long-term symptom profiles, though the predominance of dyspnea over fatigue in our cohort may reflect the higher proportion of hospitalized patients included [18,19]. International heterogeneity in symptom prevalence at 9 months is notable, with one French cohort reporting symptoms in approximately one-third of patients [20], whereas a UK cohort reported substantially higher rates [21]. Our higher prevalence likely reflects the tertiary referral nature of our institution and the inclusion of more severely ill patients.

Radiological CT findings in this cohort are consistent with prior literature. A systematic review of 67 studies reported CT abnormalities in 92.6% of COVID-19 pneumonia cases [22], and a Chinese cohort identified GGO, consolidation, and interlobular septal thickening as the most common acute patterns [23]. The persistence of GGO in 44.3% of our patients at 9 months, alongside increasing reticular opacities, pleuroparenchymal bands, and mosaic perfusion, underscores the potential for post-COVID fibrotic evolution. A UK study of hospitalized patients reported GGO in 48% at 1 year [24]. Similarly, a French study evaluating 177 patients approximately 3 months after hospital discharge reported persistent CT abnormalities in 63.2% of patients, including ground-glass opacities in 42.4% and fibrotic lesions in 19.4% [25]. In contrast, a meta-analysis of 15 studies found at least one CT abnormality in 32.6% of patients at 1 year—with critical illness associated with delayed CT resolution [26]. A meta-analysis published in *Thorax* reported that 50% of hospitalized patients had radiological inflammation at 12 months and 29% had fibrotic changes [27]. More recent evidence has similarly demonstrated that residual CT abnormalities may persist beyond 1 year after COVID-19, particularly among patients with severe acute disease [28]. The higher rates in our study may be attributable to our liberal definition of radiological significance (any single finding considered abnormal) and the inclusion of patients with pre-existing pulmonary conditions. Recent international consensus recommendations have emphasized that residual CT abnormalities may persist after COVID-19 and should be interpreted within the clinical context, taking into account the severity of the acute illness and the presence of pre-existing pulmonary disease [29].

Pulmonary function improved progressively, with significant increases in FVC and FEV1% between months 3 and 9. Although the absolute changes were modest, these improvements may be clinically meaningful because they likely reflect ongoing recovery from post-inflammatory pulmonary parenchymal injury following acute COVID-19. Reduced FVC is commonly associated with restrictive ventilatory impairment resulting from diffuse alveolar damage, organizing pneumonia, and residual interstitial abnormalities. Therefore, the observed improvement in FVC may indicate gradual resolution of inflammatory changes and partial restoration of lung compliance over time. These findings align with reports from China [30] and Spain [31], where progressive improvement in pulmonary function was similarly documented.

The predominance of restrictive patterns in ICU patients, reflected by higher FEV1/FVC ratios, is also consistent with persistent parenchymal changes after severe COVID-19 pneumonia. Furthermore, the significant improvement in 6MWT distance (*p* = 0.008) suggests recovery not only of pulmonary function but also of overall exercise capacity and functional status [32]. Since post-COVID exercise limitation may result from multiple factors, including respiratory impairment, reduced physical conditioning associated with prolonged hospitalization, cardiovascular involvement, and persistent symptoms, improvement in 6MWT performance may reflect broader recovery extending beyond pulmonary function alone [33]. Although our findings are consistent with some published series [34], other studies have reported persistent or worsening functional impairment [31], highlighting the heterogeneity of post-COVID recovery trajectories. Emerging evidence suggests that persistent pulmonary dysfunction in long COVID may be associated with ongoing inflammatory and vascular mechanisms [35].

Echocardiographic analysis revealed that ICU patients had lower EF values at 9 months compared with other groups (*p* = 0.046), suggesting persistent cardiac involvement in those with the most severe acute illness [36]. Right ventricular involvement and elevated pulmonary artery pressures have been described in the acute phase of COVID-19 [37]; long-term cardiac sequelae remain undercharacterized, and larger studies are needed to confirm these findings [38].

Diabetes mellitus was associated with delayed radiological recovery in our cohort, consistent with the known adverse effects of hyperglycemia on inflammation resolution and tissue repair. Persistent hyperglycemia has been shown to impair immune regulation, promote ongoing inflammatory activity, and negatively affect tissue healing processes, which may contribute to prolonged radiological abnormalities following COVID-19. In addition, increasing evidence suggests that SARS-CoV-2 infection may influence glucose metabolism through multiple mechanisms, including direct pancreatic β-cell injury mediated by ACE2 receptors, cytokine-induced insulin resistance, and endothelial dysfunction [39]. Recent evidence suggests that the mechanisms underlying new-onset diabetes after COVID-19 are multifactorial, involving direct viral effects, systemic inflammation, and treatment-related factors such as corticosteroid exposure [40]. Furthermore, a recent meta-analysis demonstrated an increased risk of incident diabetes following SARS-CoV-2 infection, particularly among previously hospitalized individuals. Recent studies have reported an increased incidence of newly diagnosed diabetes following COVID-19, supporting the concept that COVID-19 may have long-term metabolic consequences [41].

In our cohort, the prevalence of new-onset diabetes mellitus (3.4%) was lower than that reported by Li et al. (21%) [42] and Fadini et al. (5%) [43]. Notably, six of the seven patients with newly diagnosed diabetes had received prolonged corticosteroid therapy, suggesting that treatment-related factors may have contributed to the development of diabetes in addition to disease-related mechanisms. Nevertheless, the occurrence of new-onset diabetes during follow-up represents an important finding of our study and further supports the multisystemic nature of post-COVID sequelae. However, routine screening for diabetes mellitus was not systematically performed during follow-up in all participants. Therefore, asymptomatic cases or individuals with mild disturbances in glucose metabolism may have remained undetected, potentially leading to an underestimation of the true prevalence of new-onset diabetes in our cohort.

Pulmonary embolism was detected in 13 patients (6.34%), reinforcing data on COVID-19-associated hypercoagulability [44]. The observed prevalence in our cohort was substantially higher than expected in the general population [45]. Among the 13 patients diagnosed with pulmonary embolism, six cases were identified at the initial evaluation, six during the 3-month follow-up visit, and one during the 9-month follow-up assessment. Pulmonary embolism was investigated in patients presenting with symptoms suggestive of thromboembolic disease, particularly chest pain, rather than through routine screening. These findings emphasize the importance of considering thromboembolic complications in symptomatic patients during both the acute and follow-up phases of COVID-19.

Patients hospitalized with COVID-19 may be at increased risk of fungal infections because of multiple factors independent of SARS-CoV-2 itself, including prolonged antibiotic exposure, opportunistic pathogens, drug-related adverse effects, invasive procedures such as central venous catheterization, and critical illness [46]. Furthermore, corticosteroids and cytokine-targeted therapies, which have become integral components of the management of severe COVID-19, may contribute to immunosuppression and increase susceptibility to fungal infections [46]. In our cohort, four patients developed fungal infections during the 9-month follow-up period. Three of these patients had received prolonged corticosteroid therapy, with a mean treatment duration of 55 days, and two had a history of prolonged ICU hospitalization, with a mean ICU stay of 20.5 days. Therefore, the fungal infections observed in our cohort may have been associated not only with COVID-19 itself but also with immunosuppressive treatment and prolonged critical care exposure.

Lung cancer was diagnosed in five patients during follow-up (prevalence 2.43%). The observed cases may reflect increased detection resulting from repeated thoracic imaging, previously undiagnosed malignancies, referral bias related to the tertiary care setting, or established risk factors such as advanced age and smoking history. Although a potential association between COVID-19 and carcinogenesis has been proposed through mechanisms such as immune dysregulation and chronic inflammation triggering oncogenic processes [47], causal inference requires longer follow-up periods and larger cohorts. The relatively short follow-up period of our study further limits the ability to draw conclusions regarding a potential association between COVID-19 and subsequent cancer development.

Three patients were diagnosed with new-onset interstitial lung disease (ILD) during the 9-month follow-up. None had fibrotic abnormalities at baseline CT. Whether post-COVID fibrosing changes will progress to established ILD—and at what rate—remains an open question. Long-term data suggest that fibrotic abnormalities can persist beyond 2 years in a subset of patients [48].

### Limitations

Several limitations of this study should be acknowledged. First, this study was conducted in a tertiary referral center, which may have introduced referral bias and limited the generalizability of the findings to the broader population of COVID-19 survivors.

Second, thoracic CT examinations were performed according to clinical indications rather than a predefined imaging protocol. Therefore, patients with persistent symptoms or suspected complications may have been more likely to undergo repeat imaging, introducing potential detection bias.

Third, DLCO measurements could not be systematically performed during the study period because of technical problems affecting pulmonary function testing equipment. Since DLCO is considered one of the most sensitive indicators of post-COVID pulmonary impairment, its absence limited the comprehensive assessment of long-term pulmonary function [13].

Fourth, paired pulmonary function test data were available for only a subset of the study population. Therefore, the functional outcomes should be interpreted with caution because of the potential for selection bias.

Finally, detailed ICU-related variables, including invasive mechanical ventilation, non-invasive ventilation, high-flow nasal oxygen therapy, prone positioning, ARDS severity, and ICU-acquired complications, were not systematically collected. Consequently, the independent contribution of critical illness to long-term outcomes could not be fully evaluated [49].

## 5. Conclusions

The findings of this study support our hypothesis that COVID-19 may result in persistent clinical, functional, and radiological sequelae and that the burden of these sequelae differs according to the severity of the acute illness and hospitalization status. COVID-19 survivors, particularly those with severe acute illness, frequently exhibit persistent symptoms, radiological abnormalities, and functional impairment at 9 months. Pulmonary function and exercise capacity show improvement over time; however, some patients retain permanent pulmonary and cardiac sequelae. These findings underscore the necessity of structured, multidisciplinary long-term follow-up for COVID-19 survivors, with early identification of high-risk patients who may benefit from targeted rehabilitation and monitoring.

## Figures and Tables

**Table 1:** Demographic characteristics and acute-phase management.

Variable	*n*	%
**Age (mean ± SD, min-max)**	59.03 ± 13.6 (22–88)	
**Sex**		
Female	85	41.5
Male	120	58.5
**Hospitalization Status**		
Outpatient	63	30.7
Ward	83	40.5
ICU	59	28.8
**Treatment**		
Prednisolone	122	59.5
LTOT	53	25.9
Ward LOS (mean ± SD, days)	12.79 ± 10.0 (1–90)	
ICU LOS (mean ± SD, days)	17.49 ± 20.5 (2–120)	
**Smoking status**		
Never smoker	116	56.6
Active smoker	18	8.8
Ex-smoker	69	33.7
Pack/year (mean ± SD)	37.47 ± 29.4 (2–160)	
Comorbidity (≥1)	163	79.5
Hypertension	80	39.0
Diabetes mellitus	65	31.7
Coronary artery disease	43	21.0
COPD	11	5.4
Asthma	19	9.3
Tuberculosis	15	7.3
Malignancy	18	8.8
OSAS	8	3.9

SD: standard deviation; ICU: intensive care unit; LTOT: long-term oxygen therapy; LOS: length of stay; COPD: chronic obstructive pulmonary disease; OSAS: obstructive sleep apnea syndrome.

**Table 2:** Symptom prevalence at admission, 3 months, and 9 months.

Symptom	Admission *n* (%)	Month 3 *n* (%)	Month 9 *n* (%)	*p*
≥1 Symptom	200 (97.6)	137 (92.6)	163 (85.3)	<0.001
Dyspnea	135 (65.9)	103 (69.6)	133 (69.6)	0.419
Chest pain	37 (18.0)	45 (30.4)	62 (32.5)	0.002
Cough	105 (51.2)	51 (34.5)	68 (35.6)	0.001
Sputum	35 (17.1)	37 (25.0)	54 (28.3)	0.022
Fever	50 (24.4)	1 (0.7)	3 (1.6)	<0.001
Fatigue	65 (31.7)	31 (20.9)	32 (16.8)	0.002
Anorexia	17 (8.3)	6 (4.1)	3 (1.6)	0.006
Headache	17 (8.3)	9 (6.1)	5 (2.6)	0.040
Sore throat	19 (9.3)	3 (2.0)	1 (0.5)	<0.001
Joint pain	26 (12.7)	4 (2.7)	9 (4.7)	0.001
Memory impairment	0 (0)	6 (4.1)	13 (6.8)	0.344

**Table 3:** CT findings at admission, 3 months, and 9 months.

CT Finding	Admission *n* (%)	Month 3 *n* (%)	Month 9 *n* (%)	*p*
CT involvement	198 (96.6)	117 (92.1)	140 (79.5)	<0.001
Ground-glass opacity	185 (90.2)	86 (67.7)	78 (44.3)	<0.001
Interlobular thickening	73 (35.6)	73 (57.5)	90 (51.1)	<0.001
Reticular opacity	36 (17.6)	44 (34.6)	66 (37.5)	<0.001
Consolidation	70 (34.1)	18 (14.2)	16 (9.1)	<0.001
Mosaic perfusion	11 (5.4)	16 (12.6)	34 (19.3)	<0.001
Pleuroparenchymal band	30 (14.6)	36 (28.3)	50 (28.4)	0.001
Honeycombing	5 (2.4)	4 (3.1)	9 (5.1)	0.365
Pleural effusion	3 (1.5)	6 (4.7)	10 (5.7)	0.063
Pulmonary embolism	6 (2.9)	6 (4.7)	1 (0.6)	0.054

**Table 4:** Pulmonary function, exercise capacity, and echocardiographic findings at 3 and 9 months.

Parameter	Month 3 (*n* = 61)	Month 9 (*n* = 61)	*p*
**PFT**			
FEV1, mL (mean ± SD)	2401 ± 785.7	2509 ± 880.5	0.080
FEV1, % (mean ± SD)	82.93 ± 23.4	87.32 ± 24.8	0.026
FVC, mL (mean ± SD)	2906 ± 911.5	3064 ± 972.3	0.014
FVC, % (mean ± SD)	82.16 ± 24.1	87.03 ± 24.4	0.009
FEV1/FVC (mean ± SD)	81.40 ± 8.2	80.14 ± 7.3	0.120
6MWT, m (mean ± SD)	423.56 ± 122.7	464.10 ± 103.8	0.008
**Echocardiography**			
EF, % (mean ± SD)	56.80 ± 6.7	57.35 ± 6.9	0.322
PAP, mmHg (mean ± SD)	34.24 ± 7.9	33.42 ± 6.5	0.323
RV dilation, *n* (%)	7 (3.4)	22 (10.7)	0.301

PFT: pulmonary function test; FEV1: forced expiratory volume in 1 s; FVC: forced vital capacity; 6MWT: six-minute walk test; EF: ejection fraction; PAP: pulmonary artery pressure; RV: right ventricle; SD: standard deviation.

**Table 5:** CT findings at 9 months by acute-phase hospitalization status.

CT Finding at Month 9	Outpatient (*n* = 55) *n* (%)	Ward (*n* = 69) *n* (%)	ICU (*n* = 52) *n* (%)	*p*
CT involvement	35 (63.6)	59 (85.5)	46 (88.5)	0.003
Ground-glass opacity	20 (36.4)	33 (47.8)	25 (48.1)	0.364
Interlobular thickening	23 (41.8)	33 (47.8)	34 (65.4)	0.041
Reticular opacity	16 (29.1)	27 (39.1)	23 (44.2)	0.240
Consolidation	5 (9.1)	7 (10.1)	4 (7.7)	0.898
Mosaic perfusion	7 (12.7)	14 (20.3)	13 (25.0)	0.272
Pleuroparenchymal band	11 (20.0)	18 (26.1)	21 (40.4)	0.060

**Table 6:** New-onset conditions identified during 9-month follow-up.

New-Onset Condition	By Month 3	By Month 9
Pulmonary embolism	12	13
Diabetes mellitus	5	7
Lung cancer	2	5
Fungal infection	1	4
Interstitial lung disease	0	3
Asthma	0	4
Pneumothorax	1	3
Myocarditis	1	1
Osteoporosis	0	2
Other malignancies	0	5
Death	0	14

## Data Availability

No new data were created or analyzed in this study.

## References

[1] World Health Organization COVID-19 Dashboard. https://covid19.who.int.

[2] World Health Organization COVID-19 Vaccine Data. https://data.who.int/dashboards/covid19/vaccines.

[3] Bobrovitz N., Ware H., Ma X., Li Z., Hosseini R., Cao C., Selemon A., Whelan M., Premji Z., Issa H. (2023). Protective effectiveness of previous SARS-CoV-2 infection and hybrid immunity against the Omicron variant and severe disease: A systematic review and meta-regression. Lancet Infect. Dis..

[4] World Health Organization (2022). Severity of Disease Associated with Omicron Variant as Compared with Delta Variant.

[5] Ely E.W., Brown L.M., Fineberg H.V. (2024). Long COVID Defined. N. Engl. J. Med..

[6] Nurchis M.C., Pascucci D., Sapienza M., Villani L., D’Ambrosio F., Castrini F., Specchia M.L., Laurenti P., Damiani G. (2020). Impact of the burden of COVID-19 in Italy: Results of disability-adjusted life years (DALYs) and productivity loss. Int. J. Environ. Res. Public Health.

[7] Tang J.W., Marr L.C., Li Y., Dancer S.J. (2021). COVID-19 has redefined airborne transmission. BMJ.

[8] Tisminetzky M., Delude C., Hebert T., Carr C., Goldberg R.J., Gurwitz J.H. (2022). Age, Multiple Chronic Conditions, and COVID-19: A Literature Review. J. Gerontol. A Biol. Sci. Med. Sci..

[9] Chen N., Zhou M., Dong X., Qu J., Gong F., Han Y., Qiu Y., Wang J., Liu Y., Wei Y. (2020). Epidemiological and clinical characteristics of 99 cases of 2019 novel coronavirus pneumonia in Wuhan, China: A descriptive study. Lancet.

[10] Das K.M., Lee E.Y., Singh R., A Enani M., Al Dossari K., Van Gorkom K., Larsson S.G., Langer R.D. (2017). Follow-up chest radiographic findings in patients with MERS-CoV after recovery. Indian J. Radiol. Imaging.

[11] Graham B.L., Steenbruggen I., Miller M.R., Barjaktarevic I.Z., Cooper B.G., Hall G.L., Hallstrand T.S., Kaminsky D.A., McCarthy K., McCormack M.C. (2019). Standardization of Spirometry 2019 Update. An Official American Thoracic Society and European Respiratory Society Technical Statement. Am. J. Respir. Crit. Care Med..

[12] Mitchell C., Rahko P.S., Blauwet L.A., Canaday B., Finstuen J.A., Foster M.C., Horton K., Ogunyankin K.O., Palma R.A., Velazquez E.J. (2019). Guidelines for Performing a Comprehensive Transthoracic Echocardiographic Examination in Adults: Recommendations from the American Society of Echocardiography. J. Am. Soc. Echocardiogr..

[13] Strumiliene E., Urbonienė J., Jurgauskiene L., Zeleckiene I., Bliudzius R., Malinauskiene L., Zablockiene B., Samuilis A., Jancoriene L. (2024). Long-Term Pulmonary Sequelae and Immunological Markers in Patients Recovering from Severe and Critical COVID-19 Pneumonia: A Comprehensive Follow-Up Study. Medicina.

[14] Ko J.Y., Danielson M.L., Town M., Derado G., Greenlund K.J., Kirley P.D., Alden N.B., Yousey-Hindes K., Anderson E.J., Ryan P.A. (2021). Risk factors for COVID-19–associated hospitalization. Clin. Infect. Dis..

[15] Raimondi F., Novelli L., Ghirardi A., Russo F.M., Pellegrini D., Biza R., Trapasso R., Giuliani L., Anelli M., Amoroso M. (2021). COVID-19 and gender: Lower rate but same mortality of severe disease in women. BMC Pulm. Med..

[16] Nguyen N.T., Chinn J., De Ferrante M., Kirby K.A., Hohmann S.F., Amin A. (2021). Male gender is a predictor of higher mortality in hospitalized adults with COVID-19. PLoS ONE.

[17] Stein D.F., Manson J.J., Kerr S., Arnold D.T., Woolston A., Jackson C., Dunning J., Openshaw P.J.M., Semple M.G., Ho A. (2023). Biological sex is associated with heterogeneous responses to SARS-CoV-2 infection. Front. Immunol..

[18] Goërtz Y.M.J., Van Herck M., Delbressine J.M., Vaes A.W., Meys R., Machado F.V.C., Houben-Wilke S., Burtin C., Posthuma R., Franssen F.M.E. (2020). Persistent symptoms 3 months after a SARS-CoV-2 infection: The post-COVID-19 syndrome?. ERJ Open Res..

[19] Garrigues E., Janvier P., Kherabi Y., Le Bot A., Hamon A., Gouze H., Doucet L., Berkani S., Oliosi E., Mallart E. (2020). Post-discharge persistent symptoms and health-related quality of life after hospitalization for COVID-19. J. Infect..

[20] Zayet S., Zahra H., Royer P.-Y., Tipirdamaz C., Mercier J., Gendrin V., Lepiller Q., Marty-Quinternet S., Osman M., Belfeki N. (2021). Post-COVID-19 syndrome: Nine months after SARS-CoV-2 infection in a cohort of 354 patients. Microorganisms.

[21] Gyöngyösi M., Alcaide P., Asselbergs F.W., Brundel B.J.J.M., Camici G.G., Martins P.d.C., Ferdinandy P., Fontana M., Girao H., Gnecchi M. (2023). Long COVID and the cardiovascular system. Cardiovasc. Res..

[22] Li J., Yan R., Zhai Y., Qi X., Lei J. (2020). Chest CT findings in patients with COVID-19: A comprehensive review. Diagn. Interv. Radiol..

[23] Wu J., Wu X., Zeng W., Guo D., Fang Z., Chen L., Huang H., Li C. (2020). Chest CT findings in patients with COVID-19 and its relationship with clinical features. Investig. Radiol..

[24] Vijayakumar B., Tonkin J., Devaraj A., Philip K.E.J., Orton C.M., Desai S.R., Shah P.L. (2022). CT lung abnormalities after COVID-19 at 3 months and 1 year after hospital discharge. Radiology.

[25] Morin L., Savale L., Pham T., Colle R., Figueiredo S., Harrois A., Gasnier M., Lecoq A.-L., Meyrignac O., Noel N. (2021). Four-month clinical status of a cohort of patients after hospitalization for COVID-19. JAMA.

[26] Watanabe A., So M., Iwagami M., Fukunaga K., Takagi H., Kabata H., Kuno T. (2022). One-year follow-up CT findings in COVID-19 patients: A systematic review and meta-analysis. Respirology.

[27] Fabbri L., Moss S., A Khan F., Chi W., Xia J., Robinson K., Smyth A.R., Jenkins G., Stewart I. (2023). Parenchymal lung abnormalities following hospitalisation for COVID-19 and viral pneumonitis: A systematic review and meta-analysis. Thorax.

[28] Bocchino M., Rea G., Capitelli L., Lieto R., Bruzzese D. (2023). Chest CT Lung Abnormalities 1 Year after COVID-19: A Systematic Review and Meta-analysis. Radiology.

[29] Yoon S.H., Kanne J.P., Ashizawa K., Biederer J., Castañer E., Fan L., Frauenfelder T., Ghaye B., Henry T.S., Huang Y.-S. (2025). Best Practice: International Multisociety Consensus Statement for Post–COVID-19 Residual Abnormalities on Chest CT Scans. Radiology.

[30] Wu X., Liu X., Zhou Y., Yu H., Li R., Zhan Q., Ni F., Fang S., Lu Y., Ding X. (2021). 3-month, 6-month, 9-month, and 12-month respiratory outcomes in patients following COVID-19-related hospitalisation: A prospective study. Lancet Respir. Med..

[31] Tarraso J., Safont B., Carbonell-Asins J.A., Fernandez-Fabrellas E., Sancho-Chust J.N., Naval E., Amat B., Herrera S., Ros J.A., Soler-Cataluña J.J. (2022). Lung function and radiological findings 1 year after COVID-19: A prospective follow-up. Respir. Res..

[32] Tache-Codreanu D.L., Bobocea L., David I., Burcea C.C., Sporea C. (2024). The Role of the Six-Minute Walk Test in the Functional Evaluation of the Efficacy of Rehabilitation Programs After COVID-19. Life.

[33] Tryfonos A., Pourhamidi K., Jörnåker G., Engvall M., Eriksson L., Elhallos S., Asplund N., Mandić M., Sundblad P., Sepic A. (2024). Functional Limitations and Exercise Intolerance in Patients With Post-COVID Condition: A Randomized Crossover Clinical Trial. JAMA Netw. Open.

[34] Huang L., Yao Q., Gu X., Wang Q., Ren L., Wang Y., Hu P., Guo L., Liu M., Xu J. (2021). 1-year outcomes in hospital survivors with COVID-19: A longitudinal cohort study. Lancet.

[35] Sanhueza S., Vidal M.A., Hernandez M.A., Henriquez-Beltran M.E., Cabrera C., Quiroga R., Antilef B.E., Aguilar K.P., Castillo D.A., Llerena F.J. (2023). Clinical and pulmonary function analysis in long-COVID revealed that long-term pulmonary dysfunction is associated with vascular inflammation pathways and metabolic syndrome. Front. Med..

[36] Xie Y., Xu E., Bowe B., Al-Aly Z. (2022). Long-term cardiovascular outcomes of COVID-19. Nat. Med..

[37] Pagnesi M., Baldetti L., Beneduce A., Calvo F., Gramegna M., Pazzanese V., Ingallina G., Napolano A., Finazzi R., Ruggeri A. (2020). Pulmonary hypertension and right ventricular involvement in hospitalised patients with COVID-19. Heart.

[38] Tudoran C., Tudoran M., Lazureanu V.E., Marinescu A.R., Pop G.N., Pescariu A.S., Enache A., Cut T.G. (2021). Evidence of pulmonary hypertension after SARS-CoV-2 infection in subjects without previous significant cardiovascular pathology. J. Clin. Med..

[39] Zhao X., Jiang L., Sun W., Tang S., Kang X., Gao Q., Li Z., An X., Lian F. (2025). Understanding the interplay between COVID-19 and diabetes: Insights for the post-pandemic era. Front. Endocrinol..

[40] Kim S.H., Lee J.H. (2023). New-Onset Diabetes After COVID-19. Diabetes Care.

[41] Zilbermint M., Motevalli M., Batty K., Venner-Walcott J., Edwards A., Burley T., Jackson K., Akhtar M., Demidowich A.P. (2023). Prevalence and risk of new-onset diabetes mellitus after COVID-19: A systematic review and meta-analysis. Diabetes Res. Clin. Pract..

[42] Li H., Tian S., Chen T., Cui Z., Shi N., Zhong X., Qiu K., Zhang J., Zeng T., Chen L. (2020). Newly diagnosed diabetes is associated with a higher risk of mortality than known diabetes in hospitalized patients with COVID-19. Diabetes Obes. Metab..

[43] Fadini G.P., Morieri M.L., Boscari F., Fioretto P., Maran A., Busetto L., Bonora B.M., Selmin E., Arcidiacono G., Pinelli S. (2020). Newly-diagnosed diabetes and admission hyperglycemia predict COVID-19 severity by aggravating respiratory deterioration. Diabetes Res. Clin. Pract..

[44] Miró Ò., Jiménez S., Mebazaa A., Freund Y., Burillo-Putze G., Martín A., Martín-Sánchez F.J., García-Lamberechts E.J., Alquézar-Arbé A., Jacob J. (2021). Pulmonary embolism in patients with COVID-19: Incidence, risk factors, clinical characteristics, and outcome. Eur. Heart J..

[45] Liao S.C., Shao S.C., Chen Y.T., Chen Y.C., Hung M.J. (2020). Incidence and mortality of pulmonary embolism in COVID-19: A systematic review and meta-analysis. Crit. Care.

[46] Ulloque-Badaracco J.R., Copaja-Corzo C., Hernandez-Bustamante E.A., Cabrera-Guzmán J.C., Huayta-Cortez M.A., Carballo-Tello X.L., Seminario-Amez R.A., Hueda-Zavaleta M., Benites-Zapata V.A. (2024). Fungal infections in patients after recovering from COVID-19: A systematic review. Ther. Adv. Infect. Dis..

[47] Saini G., Aneja R. (2021). Cancer as a prospective sequela of long COVID-19. Bioessays.

[48] Li D., Liao X., Liu Z., Ma Z., Dong J., Zheng G., Zi M., Wang F., He Q., Li G. (2022). Healthy outcomes of patients with COVID-19 two years after the infection: A prospective cohort study. Emerg. Microbes Infect..

[49] Vreeman E.C.A., Pillay J., Burgess J.K. (2025). Post-COVID pulmonary sequelae: Mechanisms and potential targets to reduce persistent fibrosis. Pharmacol. Ther..

